# *In Vitro* Activity of Auranofin in Combination With Aztreonam-Avibactam Against Metallo-β-lactamase (MBL)-Producing *Enterobacterales*

**DOI:** 10.3389/fcimb.2021.755763

**Published:** 2021-10-28

**Authors:** Wen Wang, Shifeng Huang, Chunhong Zou, Yanhui Ding, Huijuan Wang, Shuli Pu, Yunfeng Liao, Hong Du, Deqiang Wang, Liang Chen, Siqiang Niu

**Affiliations:** ^1^ Department of Laboratory Medicine, The First Affiliated Hospital of Chongqing Medical University, Chongqing, China; ^2^ College of Laboratory Medicine, Chongqing Medical University, Chongqing, China; ^3^ Department of Clinical Laboratory, The Second Affiliated Hospital of Soochow University, Suzhou, China; ^4^ The Key Laboratory of Molecular Biology of Infectious Diseases designated by the Chinese Ministry of Education, Chongqing Medical University, Chongqing, China; ^5^ Hackensack Meridian Health Center for Discovery and Innovation, Nutley, NJ, United States; ^6^ Department of Medical Sciences, Hackensack Meridian School of Medicine, Nutley, NJ, United States

**Keywords:** carbapenem-resistant *Enterobacterales*, carbapenemase-producing *Enterobacterales*, metallo-β-lactamases, serine-β-lactamases, ceftazidime-avibactam, aztreonam-avibactam, auranofin, minimum inhibitory concentrations 5

## Abstract

**Objectives:**

To assess the efficacy of aztreonam-avibactam-auranofin (ATM-AVI-AUR) against a collection of 88 carbapenemase-producing *Enterobacterales* (CPE) clinical isolates and 6 *in vitro* selected ATM-AVI-resistant CPE with CMY-16 Tyr150Ser and Asn346His mutants or transformants.

**Methods:**

MICs of imipenem, ceftazidime-avibact8am (CAZ-AVI), ATM-AVI, CAZ-AVI-AUR and ATM-AVI-AUR were determined *via* the broth microdilution method. Genetic background and carbapenemase genes were determined by PCR and Sanger sequencing.

**Results:**

AUR alone showed little antibacterial activity with AUR MICs were greater than 64 μg/mL for all the 88 clinical CPE isolates. The addition of AUR (16 μg/mL) resulted in an 3-folding dilutions MIC reduction of ATM-AVI MIC_50_ (0.5 to 0.0625 μg/mL) and a 2-folding dilutions MIC reduction of MIC_90_ (1 to 0.25 μg/mL) against all 88 clinical CPE isolates, respectively. Notably, the reduced ATM-AVI MIC values were mainly found in MBL-producers, and the MIC_50_ and MIC_90_ reduced by 2-folding dilutions (0.25 to 0.0625 μg/mL) and 3-folding dilutions (2 to 0.25 μg/mL) respectively by AUR among the 51 MBL-producers. By contrast, the addition of AUR did not showed significant effects on ATM-AVI MIC_50_ (0.0625 μg/mL) and MIC_90_ (0.125 μg/mL) among single KPC-producers. Interestingly, the addition of AUR restored the ATM-AVI susceptibility against the 6 *in vitro* selected ATM-AVI-resistant CMY-16 Tyr150Ser and Asn346His mutants or transfromants, with the MICs reduced from ≥32 μg/mL (32->256 μg/mL) to ≤8 μg/mL (0.0625-8 μg/mL).

**Conclusions:**

Our results demonstrated that AUR potentiated the activities of CAZ-AVI and ATM-AVI against MBL-producing isolates *in vitro*. Importantly, AUR restored the ATM-AVI activity against ATM-AVI resistant mutant strains. As a clinically approved drug, AUR might be repurposed in combination with ATM-AVI to treat infections caused by highly resistant MBL-producing *Enterobacterales.*

## Introduction

Carbapenem resistance in carbapenem-resistant *Enterobacterales* (CRE) is driven primarily by the acquisition of various carbapenemases that are able to degrade carbapenem antibiotics. Clinically relevant carbapenemases include both serine β-lactamases (*e.g.*, KPC and OXA-48) and metallo-β-lactamases (MBLs, *e.g.*, NDM, VIM, and IMP) (Rodríguez-Baño et al., 2018). The rapid worldwide emergence of MBLs in *Enterobacterales* is especially worrisome due to their broad, constant and efficient carbapenemase activity against almost all the β-lactam antibiotics (except for monobactam, *e.g.*, aztreonam, ATM), potential for horizontal gene transfer, and the absence of clinically available inhibitors (Bush and Bradford, 2020). Although novel agents such as the newer β-lactam-β-lactamase inhibitor combinations *i.e.*, ceftazidime-avibactam (CAZ-AVI), meropenem-vaborbactam, and imipenem-relebactam, offer safe and effective therapies for serious infections caused by some of the extended-spectrum β-lactamases, KPC, AmpC, and/or OXA-48 producers, none of them present *in vitro* activity against MBL-producing *Enterobacterales* (Yahav et al., 2021). Even though the combination of ATM-AVI showed potency against MBL-producing pathogens, resistance to ATM-AVI has also been documented (Alm et al., 2015; Niu et al., 2020; Ma et al., 2020; Sadek et al., 2020).

Recent study showed that certain metallodrugs can irreversibly abrogate MBL’s carbapenemase activity *via* the displacement of Zn(II) cofactors from their active sites, suggesting a metallodrug-antibiotic combination might serve as an effective strategy to combat Zn-dependent resistant determinants, such as MBLs. The study by Wang et al. showed that an anti-peptic ulcer bismuth drug, colloidal bismuth subcitrate, could re-sensitize MBL-producers to β-lactam antibiotics through abrogating the Zn(II) cofactors by Bi(III) from the MBL active sites, thus disrupting their abilities to hydrolyze β-lactam ring in carbapenems (Wang et al., 2018). Similarly, a recent study by Sun et al. identified an antirheumatic drug, auranofin (AUR), as a potent MBL inhibitor by irreversibly abolishing MBL’s carbapenemase activity *via* the displacement of Zn(II) cofactors from their active sites. AUR demonstrated synergistic effect with imipenem (IPM) on killing a broad spectrum of CRE strains, potently restored the susceptibility of MCR-1- and NDM-5-co-producing pathogens to colistin in a murine peritonitis model, and significantly slowed down the development of carbapenem resistance (Roder and Thomson, 2015; Sun et al., 2020). In addition, a more recent study showed that AUR in combination with colistin was effective against colistin-resistent Gram-negtive bacteria both *in vitro* and *in vivo* (Feng et al., 2021).

Here we reported the activities of ATM-AVI-AUR and other antibiotics against a selection of 88 carbapenemase-producing *Enterobacterales* (CPE) clinical isolates (37 single KPC-producers, 33 single MBL-carriers, 18 dual/triple carbapenemase-producers). In addition, we investigated the impact of AUR addition on ATM-AVI activity among the *in vitro*-selected ATM-AVI highly resistant CMY-16 Tyr150Ser and Asn346His *K. pneumoniae* mutants or transformants.

## Materials and Methods

### Bacterial Strains

A total of 88 unique CPE isolates producing MBLs (*bla*_NDM-1, 4, 5, 7, 9_, *bla*_IMP-4, 8, 26_), class A carbapenemases (*bla*_KPC-2, 3)_, dual carbapenemases (*bla*_KPC/VIM_, *bla*_KPC/IMP_, *bla*_NDM/KPC_, *bla*_NDM/OXA-48-like_) or triple carbapenemases (*bla*_OXA48-like/NDM/VIM_) were selected from the archived bacterial collection from two tertiary care hospitals in Southwest (Chongqing) and Eastern China (Suzhou). We also included two *in vitro* selected ATM-AVI resistant *K. pneumoniae* CMY-16 mutants (Tyr150Ser and Asn346His) and four ATM-AVI resistant *K. pneumoniae* CMY-16 transformants harboring the Tyr150Ser and Asn346His CMY-16 pET28a plasmid constructs (Niu et al., 2020). Species were identified using a VITEK^®^ MS system (bioMérieux, France). The carbapenemase genotypes and multilocus sequence typing (MLST) were characterized by PCR and Sanger sequencing as before (Diancourt et al., 2005; Niu et al., 2020).

### Antimicrobial Susceptibility Testing

MICs for all the CPE strains were determined using standard broth microdilution method. For CAZ-AVI and ATM-AVI MICs evaluation, AVI was tested at a fixed concentration of 4 μg/mL, while CAZ and ATM were added at different concentrations ranged from 0.0625 to 256 μg/mL, respectively. For CAZ-AVI-AUR and ATM-AVI-AUR evaluation, AVI and AUR were added at the fixed concentrations of 4 μg/mL and 16 μg/mL, respectively (Wang et al., 2018), while CAZ and ATM were added at different concentrations from 0.0625 to 256 μg/mL (CLSI, 2020). Susceptibility testing was performed in triplicate on three different days. *Escherichia coli* ATCC25922 strain was used as quality control for all testing.

### *In Vitro* Selection

Multi-step selection was performed by inoculation of ~10^8^ cfu in 2-mL LB broth containing ATM-AVI at the 0.5× MICs and incubated for 24 h (Niu et al., 2020). This procedure was repeated daily, each time doubling the ATM concentration up to a maximum of 128 μg/mL, with AVI concentrations fixed at 4 μg/mL. Resistant variants were selected by plating a bacterial suspension on agar plates of corresponding ATM-AVI levels. The same multi-step selections were used to select ATM-AVI-AUR resistance, with AUR and AVI concentrations fixed at 16 and 4 μg/mL, respectively.

## Results

### Genetic Background of the CPE Isolates

Among the 88 non-duplicate clinical CPE isolates, *K. pneumoniae* was the most abundant species (*n* = 62), followed by *E. coli* (*n* = 18), *Enterobacter cloacae* (*n* = 4), *Citrobacter freundii* (*n* = 2) and *K. aerogenes* (*n* = 2). They were obtained from various clinical sources (e.g. blood cultures, *n* = 25; urine, *n* = 18; and sputum, *n* = 15), and harbored different carbapenemase genes and from different sequences types (STs). All strains had been characterized for their carbapenemase genes by PCR and DNA sequencing. The 88 clinical isolates included 37 KPC single-carbapenemase producers [KPC-2 (*n* = 36), KPC-3 (*n* = 1)], 30 NDM single-carbapenemase producers [NDM-5 (*n* = 18), NDM-1 (*n* = 8), NDM-7 (*n* = 2), NDM-4 (*n* = 1), NDM-9 (*n* = 1)]; 3 IMP single-carbapenemase producers [IMP-4 (*n* = 1), IMP-8 (*n* = 1), and IMP-26 (*n* = 1)]; 17 double-carbapenemase producers [IMP-4 and KPC-2 co-producers (*n* = 2); VIM-1 and KPC-2 co-producers (*n* = 4); NDM-1 and KPC-2 co-producers (*n* = 2); NDM-1 and OXA-181 co-producers (*n* = 2); NDM-1 and OXA-48 co-producers (*n* = 4); NDM-5 and KPC-2 co-producer (*n* = 1); NDM-5 and OXA-181 co-producers (*n* = 2); and 1 triple-carbapenemase producer with NDM-1, VIM-1 and OXA-244] (**Table 1**). MLST data showed that the 62 K*. pneumoniae* isolates belonged to 18 different ST types, with ST11 being the most common (30/62, 48.4%).

**Table 1:** MICs of CAZ-AVI-AUR and ATM-AVI-AUR against CPE isolates (μg/mL).

Strains (n)	IPM			AUR			CAZ/AVI			CAZ/AVI/AUR			ATM/AVI			ATM/AVI/AUR		
	MIC (range)	MIC_50_	MIC_90_	MIC (range)	MIC_50_	MIC_90_	MIC(range)	MIC_50_	MIC_90_	MIC(range)	MIC_50_	MIC_90_	MIC(range)	MIC_50_	MIC_90_	MIC(range)	MIC_50_	MIC_90_
All isolates (94)	**≤**0.5-256	32	128	32->64	>64	>64	0.25->128	>64	>128	≤0.0625->128	1	128	≤0.0625->256	0.5	8	≤0.004-8	0.0625	0.25
Clinical isolates (88)	**≤**0.5-256	32	128	32->64	>64	>64	0.25->128	>64	>128	≤0.0625->128	1	128	≤0.0625-8	0.5	1	≤0.004-0.5	0.0625	0.25
Single KPC-producers (37)	0.25-128	32	128	32->64	>64	>64	0.25->128	2	4	≤0.0625->128	0.25	2	≤0.0625-4	0.0625	0.125	≤0.004-0.25	0.0625	0.125
Single MBL-producers (33)	≤0.5-256	16	64	>64	>64	>64	≤0.5->128	>128	>128	≤0.0625->128	8	128	≤0.0625-2	≤0.125	1	≤0.004-0.5	0.008	0.125
Dual/triple CPE (18)	8-256	≥32	≥32	>64	>64	>64	>128	>128	>128	0.25->128	64	>128	0.0625-8	0.5	8	0.0625-0.5	0.0625	0.25
*K. pneumonia*e CMY-16 mutants & recombinants (6)	4-16	16	16	>64	>64	>64	>256	>256	>256	≤0.0625-128	16	128	32->256	128	>256	0.0625-8	1	8

IPM, imipenem; AUR, auranofin; CAZ, ceftazidime; AVI, avibactam; ATM, aztreonam.

Two CMY-16 mutants (Kp202_128A and Kp202_128B with Asn346His and Tyr150Ser, respectively) that were *in vitro* selected to be ATM-AVI-resistant and 4 CMY-16 Asn346His and Tyr150Ser *K. pneumoniae* transformant isolates (Kp214-R150, Kp231-R150, Kp214-R346 and Kp231-R346) from previous study were also included, and for ATM-AVI, MICs were interpreted according to ATM of CLSI (Niu et al., 2020; CLSI, 2020). Among them, Kp202_128B and Kp202_128A harbored CMY-16 Asn346His and Tyr150Ser substitution, respectively, showing resistance to ATM-AVI MICs (128 μg/mL). Strains Kp214-R150, Kp231-R150, Kp214-R346 and Kp231-R346 were clinical stains with the introduction of CMY-16 Asn346His and Tyr150Ser pET28a plasmid constructs, which showed increased MICs of ATM-AVI (≥ 32 μg/mL) and CAZ-ATM-AVI (≥ 16 μg/mL) (**Table 2**).

**Table 2:** Activities of ATM-AVI-AUR against ATM-AVI resistant *K. pneumoniae* isolates mutants and transformants (μg/mL).

Isolates	β-Lactamases	IPM	AUR	CAZ-AVI	CAZ-AVI-AUR	ATM-AVI	ATM-AVI-AUR
Kp202 (the parental strain)	NDM-1, OXA-48, CTX-M-15, CMY-16 (Tyr150Ser), SHV-1, TEM-1, OXA-10, SCO-1	16	>64	>256	>256	8	0.5
Kp202_128B (induced mutant)	OXA-48, CTX-M-15, CMY-16 (Tyr150Ser), SHV-1, TEM-1, OXA-10, SCO-1	4	>64	>256	0.25	>256	8
Kp202_128A (induced mutant)	OXA-48, CTX-M-15, CMY-16 (Asn346His), SHV-1, TEM-1, OXA-10	4	>64	>256	<=0.0625	128	0.0625
Kp214-R150 (recombinant)	NDM-5, OXA-181, CTX-M-15, SHV-11, TEM-1, CMY-16 (Tyr150Ser)	16	>64	>256	128	128	1
Kp231-R150 (recombinant)	NDM-1, OXA-48, CTX-M-15, SHV-11, OXA-1, CMY-16 (Tyr150Ser)	16	>64	>256	16	128	1
Kp214-R346 (recombinant)	NDM-5, OXA-181, CTX-M-15, SHV-11, TEM-1, CMY-16 (Asn346His)	16	>64	>256	128	64	8
Kp231-R346 (recombinant)	NDM-1, OXA-48, CTX-M-15, SHV-11, OXA-1, CMY-16 (Asn346His)	16	>64	>256	32	32	4

IPM, imipenem; AUR, auranofin; CAZ, ceftazidime; AVI, avibactam; ATM, aztreonam.

### *In Vitro* Activities of CAZ-AVI-AUR and ATM-AVI-AUR Against Clinical CPE Isolates

AUR alone showed little antibacterial activity with AUR MICs were greater than 64 μg/mL for all the 88 clinical CPE isolates. Susceptibility testing data showed that AUR concentrations of ≥16 μg/mL were required to reduce the ATM-AVI MICs from 8 μg/mL to <=0.5 μg/mL for all the 51 clinical MBL-positive isolates tested. And further increasing AUR concentration provided little benefit and, so, AUR was fixed at 16 μg/mL in the following *in vitro* susceptibility studies. Other agents showed various activities against these CPE isolates (**Table 1**). The majority of the isolates (84/88, 95.5%) were resistant to IPM (MIC_50 =_ 32 μg/mL), and 54.5% (*n* = 48) were resistant to CAZ-AVI (MIC_50_>64 μg/mL), none of the 88 isolates were resistant to ATM-AVI (MIC_50 =_ 0.5 μg/mL). The addition of AUR (16 μg/mL) significantly reduced the CAZ-AVI MIC_50_ from >64 to 1 μg/mL but had no significant effect on the MIC_90_ (from >128 to 128 μg/mL) in the 88 clinical CPE isolates. Compared with the result of ATM-AVI alone, the addition of AUR (16 μg/mL) resulted in an 3-folding dilutions MIC reduction of ATM-AVI MIC_50_ (from 0.5 to 0.0625 μg/mL) and a 2-folding dilutions MIC reduction of MIC_90_ (from 1 to 0.25 μg/mL), respectively.

Among the single KPC-producers (n = 37), AUR addition (16 μg/mL) did not reduce the MIC_50_ (0.0625 μg/mL) and MIC_90_ (0.125 μg/mL) of ATM-AVI, while AUR addition (16 μg/mL) reduced the MIC_50_ and MIC_90_ of CAZ-AVI from 2 μg/mL to 0.25 μg/mL (3-folding dilutions), and 4 μg/mL to 2 μg/mL (1-folding dilution), respectively.

**Figure 1:**
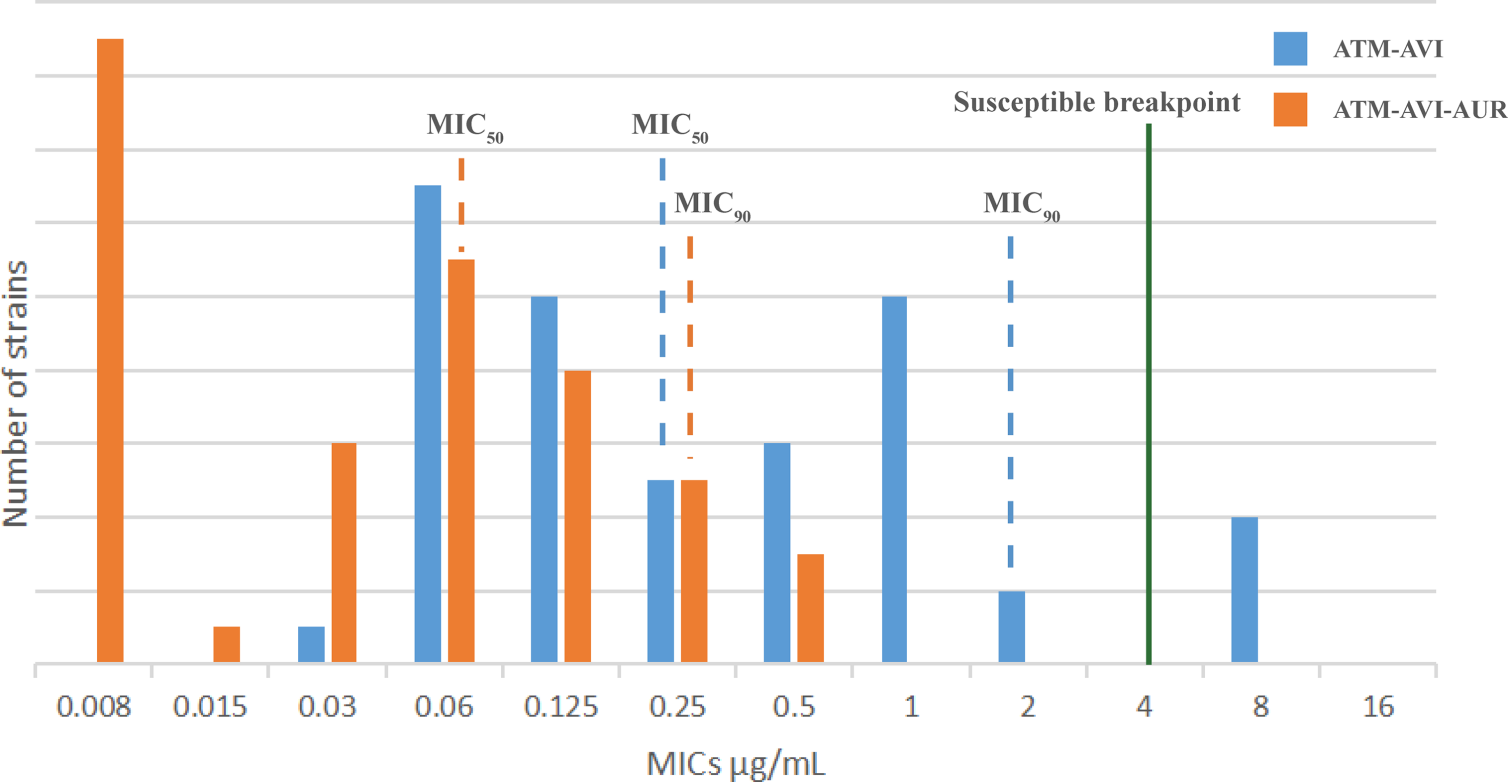
The MIC distribution of the 51 MBL-producing clinical isolates to ATM/AVI with or without AUR. The MIC_50_/MIC_90_ values of the 51 MBL-producing clinical isolates to ATM/AVI with or without AUR are shown. Isolates to the left of the green line are susceptible (for ATM-AVI, MICs are interpreted according to ATM of CLSI (2020), as there are no clinical breakpoints for ATM-AVI). With addition of AUR, the MIC_50_/MIC_90_ values of the 51 MBL-producing clinical isolates to ATM/AVI move deeper into the “susceptible zone”.

As for the MBL-producing isolates, 88.2% (45/51) isolates were highly resistant to CAZ-AVI, with MICs of CAZ-AVI >64 μg/mL. Among the 45 CAZ-AVI-resistant MBL-producing strains, the AUR addition (16 μg/mL) restore susceptibility to CAZ-AVI in 10 strains (including 7 NDM-producers, 1 IMP-producer and 2 NDM/KPC co-producers) with MICs reduced from ≥64 μg/mL to ≤4 μg/mL, however, AUR failed to sensitize CAZ-AVI resistance in remaining 35 MBL-producers (MICs≥8 μg/mL). However, the addition of AUR (16 μg/mL) significantly reduced the ATM-AVI MIC_50_ and MIC_90_ values by 2-folding dilutions (from 0.25 μg/mL to 0.0625 μg/mL) and 3-folding dilutions (from 2 to 0.25 μg/mL) respectively against the 51 clinical MBL-producers (**Figure 1**). In this collection, 4 NDM- and OXA-48/181-co-harboring clinical *K. pneumoniae* strains had the highest ATM-AVI MICs of 8 μg/mL, and the addition of AUR (16 μg/mL) reduced the MICs >4-folding dilutions (to 0.25-0.5 μg/mL).

### Activities of ATM-AVI-AUR Against ATM-AVI Resistant *K. pneumoniae* Mutants and Transformants

The six ATM-AVI resistant mutant and transformant strains all showed resistance to ATM-AVI (MIC ≥32 μg/mL), due to the presence of CMY-16 Asn346His and Tyr150Ser substitutions (Niu et al., 2020). The addition of AUR (16 μg/mL) reduced the MICs of ATM-AVI from ≥32 μg/mL (32->256 μg/mL) to ≤8 μg/mL (0.0625-8 μg/mL) in these six ATM-AVI resistant mutants or constructs (**Table 2**).

Although AUR (16 μg/mL) addition reduced the CAZ-AVI MIC by >11-folding dilutions (from >256 to 0.0625-0.25 μg/mL) in the two OXA-48-positive CMY-16 mutants (without MBLs), AUR supplementation (16 μg/mL) only led to minor MIC reductions (from >256 to 16-128 μg/mL) in the four NDM-1/NDM-5 and OXA-181-positive CMY-16 strains (**Table 2**).

In addition, our previous study showed that the CMY-16-positive, dual-carbapenemase-producing (NDM-1 and OXA-48), ST101 *K. pneumoniae* Kp202 can be *in vitro* selected to be ATM-AVI resistant (Niu et al., 2020). In this study, we examined the *in vitro* selection ATM-AVI resistance capability of Kp202 under the presence of AUR (16 μg/mL). The results showed 16 μg/mL AUR could completely inhibit the *in vitro* selected ATM-AVI resistance and no ATM-AVI-AUR resistant isolates were obtained despite multiple attempts.

## Discussion

The wide spread of multi-drug resistant Gram negative pathogens raised significant clinical and public concerns. Along with carbapenem-resistant *Acinetobacter baumannii* and carbapenem-resistant *Pseudomonas aeruginosa*, CRE top the WHO’s priority list of resistant pathogens (WHO, 2019a). CREs are also regarded as the ‘Urgent Resistance Threats’ by CDC and are prioritized in the UK’s 5-Year antimicrobial resistance national action plan (Anon, 2013; Anon, 2019). In particular, the global dissemination of MBL-producing *Enterobacterales* should be considered as the most urgent threat due to potent horizontal transfer and the lack of therapeutic inhibitors, and hence the 2019 WHO review of both the preclinical and clinical antibacterial pipeline stressed MBL-producing *Enterobacterales* as a neglected target in antibacterial drug development (WHO, 2019a; WHO, 2019b).

Therapy of invasive infections due to MBL-producers is extremely challenging, as most *bla*_MBL_-carrying plasmids co-harbor multiple additional resistance determinants, resulting in limited and sub-optimal treatment options. Although ATM is the only β-lactam antibiotic that remains stable for hydrolysis by MBLs, it can be hydrolyzed by most serine β-lactamases (SBLs), which are frequently co-produced by MBL-producers (Wu et al., 2019). AVI is a non-β-lactam-β-lactamase inhibitor which can inhibit most SBLs, but is ineffective to MBLs (Abboud et al., 2016). ATM-AVI combination has demonstrated potent *in vitro* activity against not only SBLs producers, but also MBL-producing *Enterobacterales*. One study from Sader et al. reported ATM-AVI to be highly active against 60 MBL-positive isolates (Sader et al., 2017), and another large study demonstrated potent activity of ATM-AVI against meropenem-nonsusceptible MBL-positive isolates (Karlowsky et al., 2017). A more recent study reported that ATM-AVI had potent activity against a large collection of contemporary *Enterobacterales* isolates (*n* = 8787; MIC_50_/_90_, 0.03/0.12 μg/mL), including MBL-producers (*n* = 110; MIC_50_/_90_, 0.12/0.5 μg/mL), supporting the clinical development of ATM-AVI for the treatment of infections caused by MBL-producing strains (Sader et al., 2021).

Our previous study showed that although ATM-AVI had good *in vitro* activity against MBL-producing *K. pneumoniae* isolates, clinical CMY-16-positive, dual-carbapenemase-producing (NDM-1 and OXA-48), ST101 *K. pneumoniae* strain can be successfully selected to be ATM-AVI-resistant *in vitro* (Niu et al., 2020). Worryingly, decreased susceptibilities and resistance to ATM-AVI have also been recently witnessed in clinical *E. coli* strains from India and China (Alm et al., 2015; Ma et al., 2020), due to amino-acid insertion in PBP3. Furthermore, a recent study of 118 clinical MBL-producing *E. coli* isolates from various geographical origins (Europe, Africa, Asia, and Australia) alarmingly showed 16.1% of the isolates were ATM-AVI resistant, due to a combination of PBP3 modification and the CMY-42 production (Sadek et al., 2020). These reports suggested that the clinical efficacy of ATM-AVI against MBL-producer infections could be compromised once the resistance emerges. Therefore, new drugs that specifically target MBLs and restore efficacy of last-line drugs against MBL-producing pathogens are urgently needed.

AUR directly inhibited the thioredoxin reductase (TrxR) in Gram-positive bacteria, leading to disruption of thiol-redox homeostasis and cell death. However, AUR alone does not have intrinsic antimicrobial activity against Gram-negative bacteria (Harbut et al., 2015). A recent study showed that imipenem in combination with AUR is effective against a broad spectrum of CRE strains (Sun et al., 2020). In addition, a more recent study showed that AUR in combination with colistin was effective against colistin-resistent Gram-negtive bacteria both *in vitro* and *in vivo* (Feng et al., 2021).

Here we demonstrated that AUR can potentiate the activity of both ATM-AVI and CAZ-AVI against a broad range of MBL-producing strains, and restored the activity of ATM-AVI against all the CPE clinical isolates. ATM-AVI-AUR combination was highly efficient against isolates carrying either NDM-, IMP- or VIM-type MBLs, resulting in MICs as low as 0.004-0.5 μg/mL. Our results showed that although the ATM-AVI combination is highly effective against the majority (92.16%, 47/51) of the clinical MBL-producing isolates of diverse MBLs (**Table 1**), 7.84% (4/51) of the clinical MBL-producing isolates analyzed in this study had the MICs of ATM-AVI at 8 μg/mL (intermediate for ATM based on the CLSI guideline) (CLSI, 2020). However, the addition of AUR successfully reduced the MICs of ATM-AVI by ≥4-folding dilutions, from 8 μg/mL to <=0.5 μg/mL (0.25-0.5 μg/mL). In addition, the combination of ATM-AVI-AUR showed superior effect against than that of CAZ-AVI-AUR against MBL-producers, presumably because AUR together with ATM-AVI may work synergistically to abrogate MBL enzymatic activities.

More importantly, the MICs of ATM-AVI were reduced from ≥32 μg/mL (32->256 μg/mL) to ≤8 μg/mL (0.0625-8 μg/mL) in all the 6 *in vitro* selected ATM-AVI-highly-resistant mutants and constructs with the AUR combination. Interestingly, our results also indicated that the usage of the triple combination of AUR-ATM-AVI could lower the possibility of *in vitro* selected ATM-AVI resistance. One limitation of our study is that we didn’t have any PBP3 modification caused ATM-AVI resistant strains available for testing, and it is important to examine whether ATM-AVI-AUR remain potent against these ATM-AVI resistant strain in the future.

Taken together, our study showed that AUR in combination with ATM-AVI showed potent activity against clinical CPE isolates, especially MBL-producers. ATM-AVI-AUR might provide as a therapeutic option to treat life-threatening infections caused by MBL-producing *Enterobacterales*, including strains co-producing more than one carbapenemase. Future studies are need to evaluate its *in vivo* efficacy and feasibility of clinical applications.

## Data Availability Statement

The original contributions presented in the study are included in the article/supplementary material. Further inquiries can be directed to the corresponding authors.

## Author Contributions

WW, SH, and CZ contributed to work, data analysis, and manuscript preparation. YD, HW, YL, and HD analyzed the data. SH and WW prepared the manuscript. DW, LC, and SN contributed to study design, data analysis, and manuscript preparation. All authors contributed to the article and approved the submitted version.

## Funding

This study was supported by the National Natural Science Foundation of China (Grant No. 82072349).

## Conflict of Interest

The authors declare that the research was conducted in the absence of any commercial or financial relationships that could be construed as a potential conflict of interest.

## Publisher’s Note

All claims expressed in this article are solely those of the authors and do not necessarily represent those of their affiliated organizations, or those of the publisher, the editors and the reviewers. Any product that may be evaluated in this article, or claim that may be made by its manufacturer, is not guaranteed or endorsed by the publisher.
